# Navigating through novelties concerning mCRC treatment—the role of immunotherapy, chemotherapy, and targeted therapy in mCRC

**DOI:** 10.3389/fsurg.2024.1398289

**Published:** 2024-06-14

**Authors:** Edward Zheng, Marcin Włodarczyk, Andrzej Węgiel, Aleksandra Osielczak, Maria Możdżan, Laura Biskup, Agata Grochowska, Maria Wołyniak, Dominik Gajewski, Mateusz Porc, Kasper Maryńczak, Łukasz Dziki

**Affiliations:** ^1^ Department of General and Oncological Surgery, Faculty of Medicine, Medical University of Lodz, Lodz, Poland; ^2^ Department of Biostatistics and Translational Medicine, Medical University of Lodz, Lodz, Poland

**Keywords:** metastatic colorectal cancer (mCRC), immunotherapy, chemotherapy, targeted therapy, quality of life (QoL)

## Abstract

Over the course of nearly six decades since the inception of initial trials involving 5-FU in the treatment of mCRC (metastatic colorectal cancer), our progressive comprehension of the pathophysiology, genetics, and surgical techniques related to mCRC has paved the way for the introduction of novel therapeutic modalities. These advancements not only have augmented the overall survival but have also positively impacted the quality of life (QoL) for affected individuals. Despite the remarkable progress made in the last two decades in the development of chemotherapy, immunotherapy, and target therapies, mCRC remains an incurable disease, with a 5-year survival rate of 14%. In this comprehensive review, our primary goal is to present an overview of mCRC treatment methods following the latest guidelines provided by the National Comprehensive Cancer Network (NCCN), the American Society of Clinical Oncology (ASCO), and the American Society of Colon and Rectal Surgeons (ASCRS). Emphasis has been placed on outlining treatment approaches encompassing chemotherapy, immunotherapy, targeted therapy, and surgery's role in managing mCRC. Furthermore, our review delves into prospective avenues for developing new therapies, offering a glimpse into the future of alternative pathways that hold potential for advancing the field.

## 1 Introduction

CRC (colorectal cancer) is one of the most common cancers among all populations accounting for nearly one-third of all diagnosed cancers worldwide according to WHO and is characterized by high lethality with an approximately 14% 5-year survival rate (1). Median age of CRC diagnosis according to (2) 70–72 years while approximately 19%–22% (3, 4) of patients are found to have already progressed to metastatic colorectal cancer (mCRC) at the time of initial diagnosis.

Like every neoplastic disease, CRC is inherently a genetic disorder. This stems from the nature of the colon and rectum, where the epithelium undergoes frequent turnover and is subject to damage due to exposure to chemical and biological substances which can lead to sporadic mutations within genes categorized as tumour suppressor genes (TSGs) such as APC, DCC, TP53, SMAD2, SMAD4, and p16INK4a, proto-oncogenes such as BRAF, K-ras, N-ras, as well as genes involved in DNA repair mechanisms such as MMR and MUTYH (5) subsequently leading to malignancy. The second group of patients who develop CRC independently of environmental factors consists of individuals burdened with hereditary syndromes, which predispose individuals to either polyposis conditions, such as familial adenomatous polyposis (FAP), or syndromes without polyposis (HNPCC) but associated with microsatellite instability, such as Lynch syndrome.

The management of regional and localized CRC has been practised with varying success since the 19th and 20th centuries (6). However, the introduction of chemotherapy marked a pivotal advancement by offering a chance for life extension to patients diagnosed with mCRC. Initially, 5-fluorouracil-based treatments showed significant improvement in overall survival (OS), extending it to 14 months. Further enhancements, such as incorporating leucovorin and oxaliplatin, raised OS to 19.5 months (7). Despite nearly 60 years of optimizing chemotherapy since its introduction, its role remains mostly limited to palliative care, and it still carries side effects and questionable efficacy for certain patients (8). As such current standard care for mCRC is largely personalized and encompasses surgical resection, including complete removal of primary tumour and metastases in the liver, lung, and peritoneal, and personalized systemic therapy comprising chemotherapy, targeted therapy, and immunotherapy which yields a notable increase of OS of around 2 years. According to ASCO guidelines, personalization is based mostly on the status of microsatellite stability, mismatch repair, RAS mutations, and the location of the primary tumour (left-sided or right-sided tumour). Among all individuals with previously untreated, initially unresectable mCRC doublet chemotherapy or alternatively triplet regimen combined with anti-vascular endothelial growth factor (anti-VEGF) antibodies should be considered as a first-line treatment.

However, if the patient additionally exhibits microsatellite instability-high (MSI-h) and/or deficient mismatch repair (dMMR) immunotherapy involving anti-CTLA-4 or anti-PD1 agents should be considered. For patients who present microsatellite-stable (MSS) or proficient mismatch repair left-sided treatment-naive RAS wild-type mCRC, a combination of chemotherapy and anti-epidermal growth factor receptor (anti-EGFR) therapy is highly recommended, meanwhile, among microsatellite-stable (MSS) or proficient mismatch repair (pMMR) RAS wild-type right-sided mCRC treatment with chemotherapy and anti-vascular endothelial growth factor therapy should be considered.

Regardless of persistent challenges in managing mCRC, the past 20 years were marked with unprecedented progress in genetics, cellular physiology, and the immune system have catalyzed the development of a myriad of innovative therapeutic methods, promising an increasingly more favourable prognosis and QoL among patients with mCRC such as immunotherapy based on CTLA-4 and PD-1 pathway inhibition, or targeted therapy focused on specific receptors or ligands e.g., VEGF and EGFR.

## 2 The role of the immunotherapy in the mCRC management

**Figure 1:**
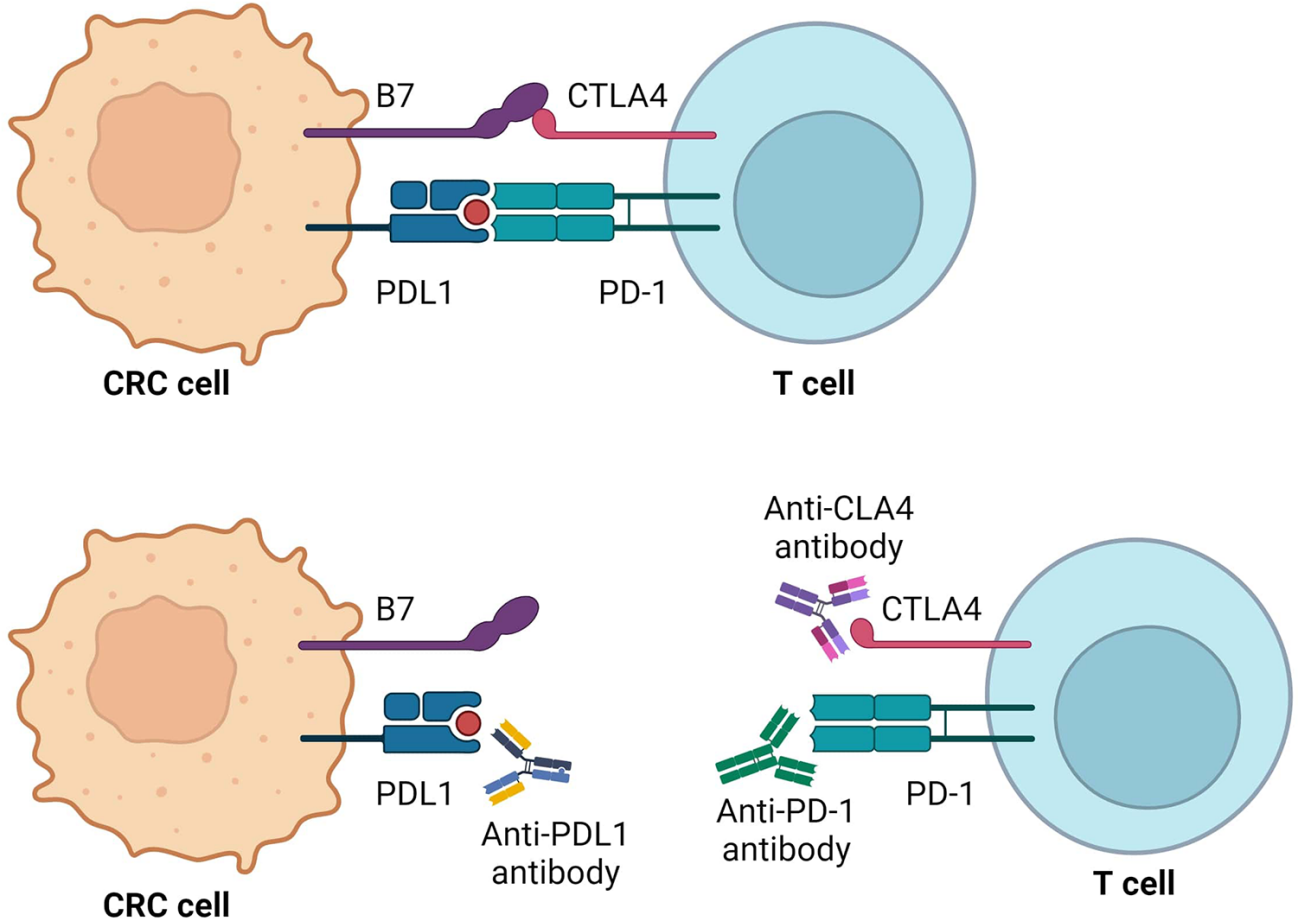
PD-1 and CTLA-4 pathway-mediated inhibition of immune response and mechanisms of action of immunotherapy agents. Created with BioRender.com.

The fundamental framework of the anti-tumour response by the immune system primarily relies on cellular immunity, in which the activation of cytotoxic T cells (CD8+) occurs through the cross-presentation of tumour-associated antigens (TAA) or neoantigens by dendritic cells (Figure 1) (9). Clinically, mCRC is characterized by a weak immune response, attributed to the observation that mCRC exhibits fewer somatic mutations, represented by proficient mismatch repair (pMMR) and/or microsatellite stability (MSS), compared to other cancers which result in diminished immunogenicity (10, 11). Additionally, other factors, such as the lack of tumour antigen presentation, insufficient T lymphocyte penetration, or T cell suppression by the tumour, may also influence the competency of the immune system in mCRC (12).

Contemporary immunotherapy primarily focuses on inhibiting immune checkpoints (ICP), notably cytotoxic T-lymphocyte–associated antigen 4 (CTLA-4) and programmed death 1 (PD-1), which act as down-regulators of cytotoxic T-cell immunity. Inhibiting these pathways activates the immune system, promoting a cytotoxic effect on tumour cells (13). Moreover, immunotherapy exhibits lower toxicity on normal cells (14) reducing the occurrence of side effects compared to standard chemotherapy. Common adverse effects include dermatological symptoms such as rash and pruritus, along with diarrhoea and colitis developing within 6–8 weeks of treatment onset. Approximately one-fifth of patients experience elevated hepatic enzyme levels, and some report fatigue, nausea, and headache. Toxicities are more frequent with anti–CTLA–4 inhibition, while anti-PD-–PD–1 checkpoint inhibitors show slightly higher rates of ADRs (15, 16).

The rationale for the widespread use of immunotherapy in mCRC across all patient groups, regardless of tumour mutational burden (TMB) status, remains unclear. Notably, tumours with pMMR, MSS, or low microsatellite instability (MSI-L) exhibit resistance to immune checkpoint inhibitors (ICI) due to low TMB and a deficiency in immune cell infiltration (12). Furthermore, the subset of patients with dMMR or MSI-H, who may potentially benefit from ICI therapy, constitutes a relatively limited proportion, approximately 15% of CRC patients (17, 18). Additionally, those patients who initially respond to immunotherapy often swiftly progress into a state of immune resistance (19).

Despite the limitations of immunotherapy, the prospect of combination therapies, integrating immunotherapy with chemotherapy, radiotherapy, or a second immunotherapy agent, has demonstrated promising results in trials such as CCTG CO.26 (20), MAYA (21) and atezoTRIBE (22).

### 2.1 PD-1 inhibitors

The Programmed Cell Death Protein 1 (PD-1), a crucial member of the checkpoint proteins and a member of the CD28 family (23), plays an important role in immune regulation. Its ligand, Programmed Death-Ligand 1 (PDL-1), is a type 1 transmembrane glycoprotein belonging to the B7 ligand family and is primarily expressed on the surface of tumour cells (24). The PD-1/PDL-1 pathway is activated in response to infections, serving to restrain the elimination of host cells and mitigate the risk of autoimmune diseases (25). In the context of malignancies, the PD-1/PDL-1 signal transduction pathway assumes one of the central roles in the mechanisms of tumour-mediated immunosuppression which facilitates immune evasion by suppressing the anti-tumour immune response (26, 27).

The FDA has approved several monoclonal antibodies targeting PD-1, including nivolumab, pembrolizumab, cemiplimab, dostarlimab, retifanlimab, and toripalimab. Additionally, three PDL-1 inhibitors, namely atezolizumab, durvalumab, and avelumab, have also received FDA approval. Immunotherapy has durably demonstrated efficacy in the treatment of various haematological and solid malignancies such as melanoma, non–small cell lung cancer, renal cell carcinoma, urothelial carcinoma, gastroesophageal carcinoma, and hepatocellular carcinoma have seen notable clinical benefits from PD-1/PDL-1 blockade (See Table 1) (28, 29). In case of mCRC, pembrolizumab and nivolumab are among the FDA-approved PD-1/PDL-1 inhibitors (30).

**Table 1:** Overview of landmark trials involving immunotherapy agents for mCRC management.

Trial	Phase	Characteristics of the studied population	Number of participants	Treatment	Primary Endpoint	Results
KEYNOTE-016	II	mCRC with or without mismatch-repair deficiency	11	Pembrolizumab	ORR PFS at 20 weeks	dMMR mCRC vs. pMMR mCRC: ORR: 40% vs. 0% PFS: 78% vs. 11%
KEYNOTE-164	II	Previously treated patients with MSI-H/dMMR mCRC	124	Pembrolizumab	ORR	≥ 2 prior line of therapy vs. ≥1prior line of therapy: ORR: 33% vs. 33% PFS at 24 months: 31% vs. 37% OS at 24 months: 55% vs. 63%
KEYNOTE-177	III	First-line therapy for MSI-H/dMMR mCRC patients	307	Pembrolizumab vs. standard treatment	OS PFS	Pembrolizumab vs. standard treatment The PFS at 24 months rates: 48.3% vs. 18.6% ORR: 43.8% vs. 33.1% OS HR 0.74, 95% CI 0.53–1.03
CheckMate-142	II	Recurrent or metastatic MSI-H/dMMR CRC	45	Nivolumab + Ipilimumab/	ORR	ORR: 69% DCR: 89% PFS at 24 months: 74%
CheckMate-142	II	Previously treated patients with MSI-H/dMMR mCRC	119	Nivolumab + Ipilimumab	ORR	ORR: 55% at 13 months to 65% at 51 months, DCR: 81% at 51 months
CheckMate-142	II	Previously treated patients with MSI-H/dMMR mCRC	74	Nivolumab	ORR	ORR: 31% DCR at 12 weeks: 69% PFS at 12 months: 51%
NICHE	II	CRC patients with dMMR or pMMR	40	Ipilimumab + Nivolumab	PR	dMMR vs. pMMR: PR: 100% vs. 27%
GERCOR NIPICOL	II	Previously treated patients with MSI-H/dMMR mCRC	57	Nivolumab + Ipilimumab	DCR at 12 weeks	DCR at 12 months: 86% ORR: 60% PFS at 12 months: 73% OS rate at 12 months: 84%
BACCI	II	mCRC patients with or without mismatch-repair deficiency	133	Capecitabine, bevacizumab (CB) with or without atezolizumab	PFS	CB with: placebo vs. artezolizumab PFS: 3.3 months vs. 4.4 months The OS at 12 monts: 43% vs. 52%
MAYA	II	mCRC patients with MSS status and MGMT silencing	708	Nivolumab + Ipilimumab	PFS at 8 months	PFS at 8 months: 32% median OS: 18.5 months ORR: 39%
AtezoTRIBE	II	Previously treated patients with mCRC	218	Standard treatment with or without atezolizumab	PFS	Standard treatment vs. standard treatment + atezolizumab PFS: 11.5 vs. 13 months median OS: 27 vs. 33 months
CCTG CO.26	II	Previously treated patients with mCRC	180	Durvalumab + tremelimumab vs. Best Supportive Care (BSC)	OS	Durvalumab + tremelimumab vs. BSC median OS: 6.6 vs. 4.1 months median PFS: 1.8 vs. 1.9 months DCR: 22.7% vs. 6.6%
SAMCO-PRODIGE 54	II	Previously treated patients with MSI-H/dMMR mCRC	122	Avelumab vs. Standard Second-Line Chemotherapy	PFS	Avelumab vs. Standard Second-Line Chemotherapy PFS at 12 months: 31.2% vs. 19.4% PFS at 18 months: 27.4% vs. 9.1% ORR: 29.5% vs. 26.2% DCR at 18 months: 75.7% vs. 19.1%

### 2.2 CTLA-4 inhibitors

Cytotoxic T-lymphocyte antigen-4 (CTLA-4), predominantly expressed on the surface of activated T cells, serves as an immune checkpoint involved in the early-stage response of T cells. In contrast to the stimulatory signal mediated by CD28, CTLA-4 transduces an inhibitory signal to T cells, regulating the delicate balance of immune responses (31). The dynamic interplay between CTLA-4 and CD28 occurs through their interaction with B7 ligands expressed on the surface of antigen-presenting cells. Notably, CTLA-4 exhibits a 20-fold higher affinity for the B7 ligand compared to CD28, resulting in a potent inhibitory effect on T cells which contributes to the suppression of T cell activation and proliferation (32). The competition for B7 ligands between CD28 and CTLA-4 establishes a regulatory mechanism in which CTLA-4 dampens T-cell responses. The mediatory role of CTLA-4 extends to the inhibition of T-lymphocyte responses, leading to the curtailment of T lymphocyte proliferation. Furthermore, CTLA-4 promotes the heightened activity of regulatory T cells (Tregs), further contributing to immune tolerance (33).

FDA-approved therapies targeting CTLA-4 in combination with PD-1/PDL-1 inhibitors have shown promise in the treatment of mCRC. Combining anti-CTLA-4 therapy with PD-1/PDL-1 blockade represents a synergistic approach to enhance the anti-tumor immune response. While combination therapy has gained regulatory approval, the efficacy of monotherapy with CTLA-4 inhibitors is still under evaluation (34).

### 2.3 Immunotherapy agents

#### 2.3.1 Pembrolizumab

Pembrolizumab is a humanized monoclonal anti-PD1, approved primarily for the management of melanoma in September 2014 by the FDA (35), currently being utilized for the treatment of a plethora of cancers including melanoma, small-cell lung cancer, Hodgkin lymphoma, urogynaecological and gastrointestinal cancers (28) as far as the role of pembrolizumab in the management of CRC and mCRC is concerned the KEYNOTE series, particularly KEYNOTE-016, KEYNOTE-164, KEYNOTE-012, KEYNOTE-028, and KEYNOTE-177, played a significant role in investigating the role of PD-1 inhibitor pembrolizumab for the mCRC and CRC treatment. The trials focused on patients with DNA mismatch repair and microsatellite stability status, revealing promising outcomes in terms of response to PD-1/PD-L1 inhibitor therapy among mCRC patients (12). The initial phase I trial, KEYNOTE-028, while not demonstrating substantial advantages in OR among CRC patients, provided an insight into the safety profile of pembrolizumab. Despite a meagre response rate (1 out of 23 patients), the treatment exhibited a favourable safety profile, with only 35% of the cohort reporting ADRs. Following the profiling of a sole responder in KEYNOTE-028, researchers began to suspect the potential role of MSI in treatment efficacy (36). Subsequent phase 2 trials, including KEYNOTE-016, KEYNOTE-164, KEYNOTE-012, and KEYNOTE-158, corroborated the suspected link between MSI-H, dMMR, and a positive response to pembrolizumab treatment among CRC patients.

Additionally, KEYNOTE-158 revealed a correlation between high tumour mutation burden (TMB) and improved overall response to ICB therapy (37). The compelling results from these trials led to the FDA approval of pembrolizumab for adult and pediatric patients with unresectable or metastatic MSI-H or dMMR solid tumours that have progressed after prior treatment, with no satisfactory alternative options. However, the subsequent phase 3 study, KEYNOTE-177, designed to assess OS in patients with dMMR or MSI-H mCRC, did not demonstrate the superiority of pembrolizumab as a first-line treatment over standard chemotherapy combined with an anti-VEGF agent. Despite the lack of OS superiority, the pembrolizumab regimen exhibited a more favourable PFS compared to chemotherapy (median PFS of 16.5 months vs. 8.2 months).

#### 2.3.2 Nivolumab and ipilimumab

In 2016, the initial trial under the acronym CHECKMATE was initiated to investigate the potential role of nivolumab, a fully human IgG4 PD-1 immune checkpoint inhibitor antibody (38), in various malignancies, including squamous cell lung cancer, melanoma, and hepatic cancer. The studies within the CHECKMATE-142 series specifically assessed the efficacy of nivolumab in treating advanced MSI-H CRC, either as monotherapy or in combination with ipilimumab, a CTLA-4 inhibitor (39).

CHECKMATE- 142, a multicenter multicohort non-randomized study designed to evaluate the efficacy and safety of nivolumab either as monotherapy or in combination with ipilimumab, a CTLA-4 inhibitor, in advanced MSI-H CRC patients. The study reported an ORR of 31% and a DCR of 68% with nivolumab in a monotherapy regime, however, the dual therapy with ipilimumab resulted in superior outcomes, with ORR and DCR exceeding 60% and 84%, respectively. Also, in combination therapy, the PFS rate was estimated to be 77% and the OS was 83% in the 1 year. In comparison, patients undergoing nivolumab monotherapy exhibited a PFS of 50% and an OS of 73% at the same 1-year interval. Due to notable efficacy and safety demonstrated during the CHECKMATE-142 trial (40), the FDA eventually approved nivolumab as a single agent or in combination for treating MSI-H mCRC in 2017.

Importantly, the response was observed across all patient subgroups, irrespective of tumour PD-L1 expression and regardless of clinical history, including those with KRAS or BRAF mutations. In patients with BRAF V600E mutations, an ORR of 25% was observed, surpassing rates with traditional chemotherapy or combination therapy with BRAF EGFR or MEK inhibitors. These findings suggest that nivolumab may exhibit superior activity compared to conventional therapies in patients with BRAF-mutant mCRC, a subgroup typically associated with a poor prognosis and MSI-h mCRC.

The acknowledgment of the limitation of the non-RCT highlighted the need for an ongoing randomized phase III CheckMate-8HW trial (41), in recurrent or metastatic MSI-H/dMMR CRC. The efficacy of three therapeutic approaches is currently under evaluation: a combination of nivolumab and ipilimumab, nivolumab alone, and chemotherapy.

The utilization of ipilimumab and nivolumab was additionally employed for the treatment of mCRC with MMS status, concomitantly with chemotherapy. The phase II MAYA trial (21) investigated the impact of temozolomide on TMB, concerning the response to immune checkpoint inhibitors (ICI) therapy in patients with MSS mCRC. Eligible patients underwent two priming cycles of temozolomide followed, in the absence of disease progression, by the introduction of a combination of ipilimumab and nivolumab. Out of 135 patients, 33 reached the second treatment phase. The primary endpoint of the PFS rate at 8 months was achieved, recording a rate of 36%. Median PFS and OS were 7 and 18 months, respectively, and an ORR of 45% was observed, indicating delayed or gradual responses consistent with the efficacy of immunotherapy. Moreover, all ADRs of the combinations were easily manageable. The MAYA study substantiated the concept that a sequential regimen involving temozolomide priming, followed by a combination of ipilimumab and nivolumab, suggests that combining therapy may help overcome drug-resistant patients mCRC with MSS.

#### 2.3.3 Durvalumab

Durvalumab received approval from the FDA in 2017 for the treatment of locally advanced or metastatic urothelial carcinoma. Currently, its therapeutic applications extend to non-small cell lung cancer (42). In combination with chemotherapy, durvalumab is employed in the management of extensive-stage small-cell lung cancer and biliary tract cancer (43).

Durvalumab's potential as a therapeutic option for mCRC patients is under evaluation, with a focus on clinical trial data and safety profiles. In the phase 2 trial led by (44), durvalumab monotherapy exhibited promising antitumor activity and maintained a manageable safety profile in patients with previously treated mCRC with MSI-H/dMMR variant or with POLE mutation. The PFS rate in those patients at 12 months was determined to be 58.2% (95% CI, 39%–73%), and the 12-month OS rate was 68.3% (95% CI, 49%–82%). These outcomes suggest that durvalumab monotherapy yields results comparable to the clinical efficacy observed with already approved PD-1 inhibitors such as pembrolizumab and nivolumab. The findings from this study contribute valuable insights into the potential of durvalumab as a therapeutic option in those mCRC patients.

Durvalumab has also been trialled in combination with tremelimumab and mFOLFOX6 chemotherapy regime among RAS mutated unresectable mCRC during phase 1b/2 MEDITREME trial (45). In conclusion, in the phase 2 study, the primary objective of achieving a 3-month PFS was met by 90.7% of patients with MSS mCRC. Secondary objectives revealed notable outcomes 6 (95.8%), 12 (81.1%), 24 (57.6%) months OS, mPFS (8.2%), RECIST (52%), and DCR (93.7%). Furthermore, in the course of the trial, three colorectal cancer (CRC) patients with mutations in the POLE gene exhibited a response to durvalumab. Notably, one patient with a mutation in the exonuclease region demonstrated an objective response, while those with mutations outside this region experienced disease progression which might suggest a potential role of the mutations in the exonuclease region of the POLE gene in the treatment response.

Another randomized phase II trial centred on evaluating the combination of durvalumab and tremelimumab—CCTG CO.26 (20) has provided convincing evidence that double ICI therapy yields superior outcomes as the OS in patients treated with durvalumab plus tremelimumab, with 166 out of 180 patients defined as MSS/pMMR, reached 6.6 months in comparison to the 4.1 months of OS observed in patients receiving best supportive care (BSC).

#### 2.3.4 Avelumab

In March 2017, the FDA granted approval for avelumab in the therapeutic management of patients with metastatic Merkel-cell carcinoma (46). Subsequently, the scope of avelumab applications has been expanded to encompass the treatment of advanced or metastatic renal cell carcinoma and urothelial carcinoma (47, 48).

Various trials are currently being conducted to evaluate the effect of treatment on the development of mCRC. The phase I clinical trial, conducted by (49) focused on evaluating safety and response in 22 patients with mCRC. The results of this trial proved the safety profile of avelumab to be consistent with that of other monoclonal antibodies anti-PD-1/PD-L1, however indicated that the drug lacked efficacy in this group of patients, as mPFS was 2.1 months (95% CI: 1.4–5.5 months) and none of the patients produce objective responses to the avelumab. Its lack of effectiveness stemmed from the fact that the trial did not categorize mCRC with MSI and MSS.

The phase 2 randomized clinical trial, 2 SAMCO-PRODIGE 54 (50), included 122 patients, who were randomized to receive avelumab or standard second-line therapy (chemotherapy). Avelumab compared to chemotherapy was associated with significantly better PFS at 12 months and 18 months (31% vs. 19%; 27% vs. 9%, respectively). In addition, avelumab had a favourable safety profile in the second-line therapy of dMMR/MSI mCRC, as the avelumab group demonstrated a lower incidence of treatment-related adverse events compared to the chemotherapy group (32% vs. 34%). ORR (30% vs. 26%) and DCR (71% vs. 77%) were similar between those cohorts.

#### 2.3.5 Atezolizumab

In a phase 2 clinical trial of AtezoTRIBE (51), 218 patients with previously untreated mCRC were examined to evaluate the effect of combining first-line FOLFOXIRI plus bevacizumab with the anti-PD-L1 agent—atezolizumab.

With a median follow-up duration of 20 months, the atezolizumab group demonstrated a mPFS of 13 months (80% CI 12.5–13.8), compared to 12 months (10.0–12.6) of mPFS exhibited by the control group receiving only first-line FOLFOXIRI plus bevacizumab. Nevertheless, the hazard ratio was 0.69 (80% CI 0.56–0.85) with a statistically significant *p*-value of 0.012.

However, IMblaze370, a randomized phase III trial (52) revealed diminished efficacy of atezolizumab in mCRC management when compared to regorafenib, as evidenced by a mOS of 7.1 months vs. 8.5 months, respectively. The combination of atezolizumab and cobimetinib demonstrated an OS of 8.9 months. The ORR was estimated at 2.2%, 2.2%, and 2.7% for atezolizumab monotherapy, regorafenib, and atezolizumab with cobimetinib, respectively.

The study outcomes indicate that neither immune checkpoint inhibitor monotherapy nor its combination with targeted therapy succeeded in improving the DFS and OS in mCRC patients.

Moreover, the phase II randomized study—BACCI (53), demonstrated a weak signal of efficacy with adding atezolizumab to capecitabine and bevacizumab in mCRC management. Patients, who received atezolizumab in conjunction with capecitabine and bevacizumab (ACB) exhibited a mPFS of 4,4 months, whereas patients, who received a placebo to capecitabine, and bevacizumab (PCB) had a mPFS of 3,3 months [an HR of 0.725 (0.491–1.07, *p* = 0.051]. The ORR and OS were 4.4% vs. 8.5%, *p* = 0.5, in PCB and ACB, respectively. The OS at 12 months was 43% vs. 52% [HR 0.94 (0.56–1.56), *p* = 0.4] in PCB and ACB, respectively.

#### 2.3.6 Dostarlimab

According to a recent clinical trial referenced as NCT04165772, dostarlimab has proven to be an incredibly effective treatment for dMMR rectal cancer. Out of the 12 patients who participated, all achieved a clinical complete response rate of 100% (95% CI, 74%-100%) (54). During the six-month treatment period, none of the participants reported any adverse events of grade 3 or higher. Most patients experienced rapid relief of disease symptoms within nine weeks of initiating dostarlimab, with 81% reporting resolution. Endoscopic complete response was observed in 42% of patients, and 15% achieved a radiographic complete response to this anti-PD-1 therapy.

#### 2.3.7 Retifanlimab

Retifanlimab is one of the most recently approved PD-1 inhibitors that shows promise in treating patients with metastatic or recurrent locally advanced Merkel cell carcinoma (55). However, there is currently no research on the effects of retifanlimab on mCRC. While one study evaluating a personalized neoantigen vaccine combined with retifanlimab has been withdrawn (NCT04799431), another study testing the effectiveness of a combination of retifanlimab, TriAdeno vaccine, N-803, and SX-682 is still in the preparatory process and has not yet begun recruitment (NCT06149481).

## 3 Targeted therapy

Targeted therapy in cancer treatment involves the use of drugs that specifically target certain biological features of tumour cells rather than non-specific neutralization (56). This approach has gained significant momentum in the treatment of mCRC since the approval of the first targeted drug, cetuximab, by the FDA in 2004. Cetuximab, an anti-EGFR IgG1 antibody, marked a pivotal moment in the field, paving the way for a deeper understanding of cellular pathways and the development of agents that target specific pathways implicated in cancer progression.

**Figure 2:**
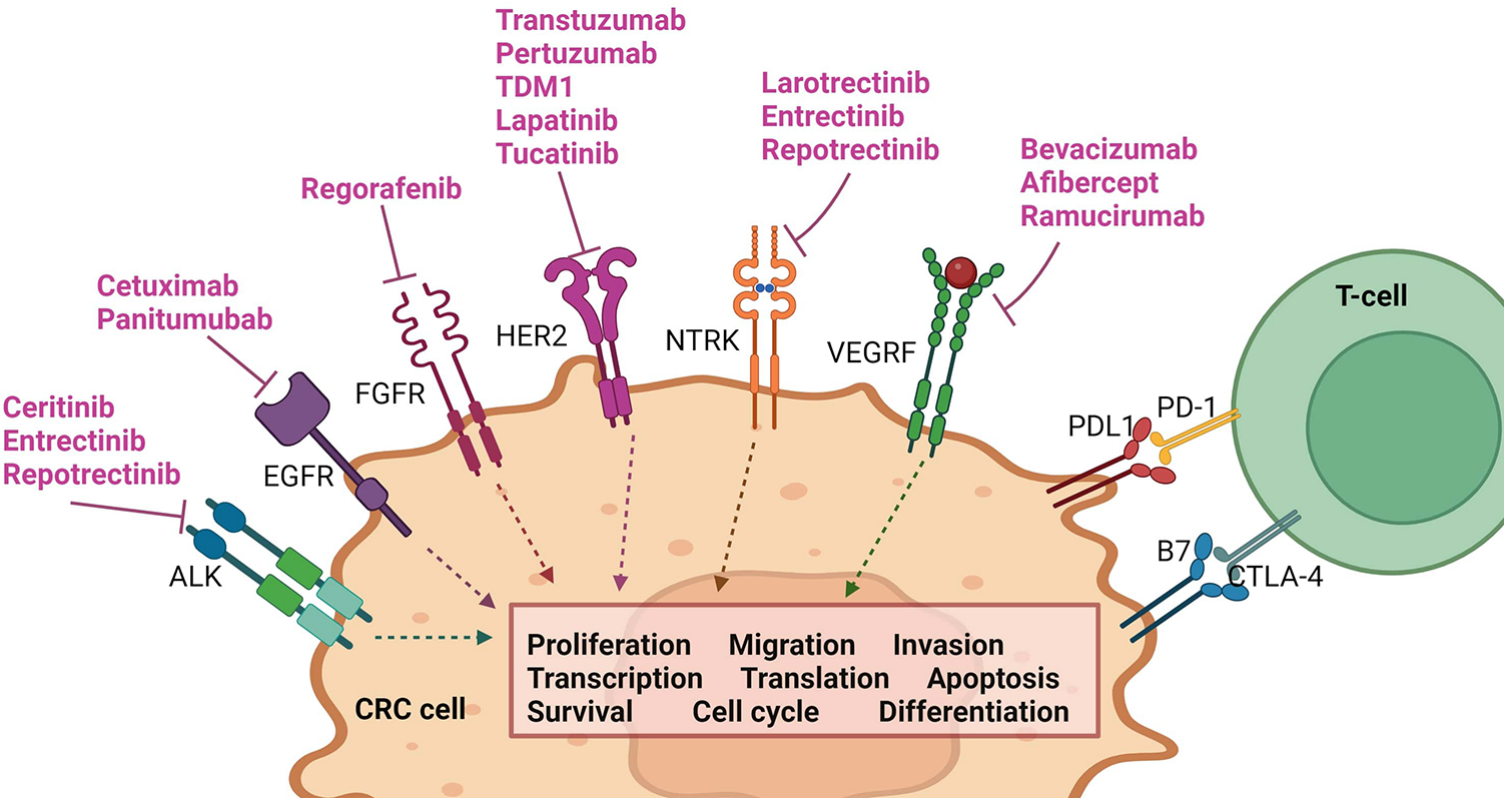
Pathways involved in common targeted therapy currently utilized in mCRC management. Created with BioRender.com.

**Figure 3:**
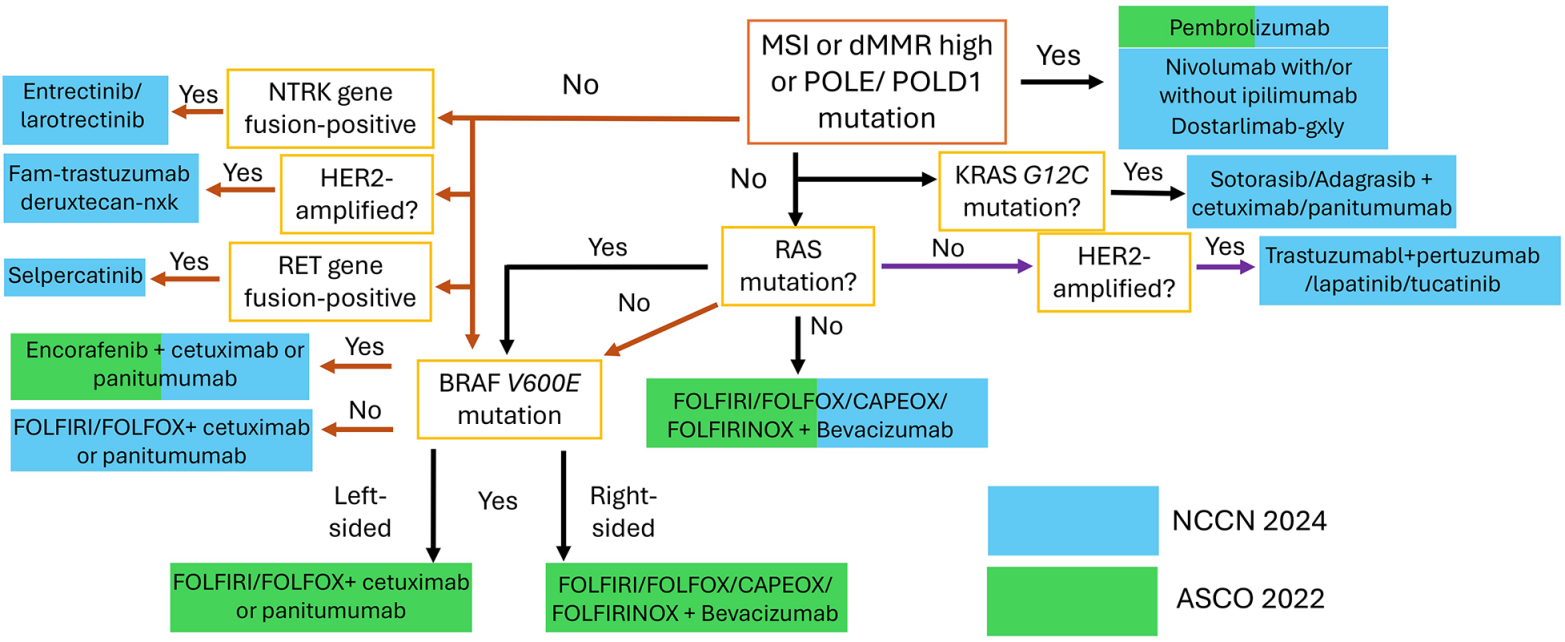
Overview of the latest NCCN and ASCO guidelines regarding the biomarker-based stratification for first-line treatment of mCRC.

Noteworthy pathways include those involving epidermal growth factor receptor (EGFR), fibroblast growth factor receptor (FGFR), vascular endothelial growth factor receptor (VEGF-R), and tropomyosin receptor kinase (TRK) (see Figure 2). The elucidation of these pathways has led to the development of a variety of agents that selectively target them. These targeted therapies have undergone extensive testing and have been integrated into clinical practice, contributing significantly to the improvement of QoL and life expectancy for specific groups of patients with mCRC (see Figure 3 and Table 2) (8, 56, 57).

**Table 2:** Overview of landmark trials involving targeted therapy agents for mCRC manage.

Trial	Phase	Characteristics of the studied population	Number of participants	Treatment	Primary endpoint	Endpoint results
PRIME	III	wild-type (WT) KRAS mCRC	1,183	Panitumumab	PFS	FOFLOX4 + panitumumab vs. FOLFOX4 (10 vs. 8.6 months)
CRYSTAL	III	WT KRAS mCRC	1,198	Cetuximab	PFS	Best supportive care + panitumumab vs. Best supportive care (8 vs. 7.3 weeks)
ASPECCT	III	WT KRAS mCRC	999	Panitumumab vs. cetuximab	OS	panitumumab vs. cetuximab (10.4 vs. 10 months)
Fire-3	III	KRAS exon 2 WT mCRC	592	Cetuximab vs. bevacizumab to FOLPIRI	ORR	FOLFIRI plus cetuximab vs. FOLFIRI plus bevacizumab (62% vs. 58%)
SWOG S1406	II	BRAFV600E-mutated mCRC	106	Irinotecan, cetuximab and vemurafenib	PFS	Irinotecan + Cetuximab + Vemurafenib vs. Irinotecan + Cetuximab (4.2 vs. 2 months)
PANAMA	II	RAS WT mCRC	248	Panitumumab, folinic acid, fluorouracil	PFS	FU/FA + panitumumab vs. FU/FA (8.8 vs. 5.7 months)
Napolitano et al	II	RAS WT mCRC	62	Panitumumab, tifluridine-tipiracil	PFS	trifluridine-tipiracil + panitumumab vs. trifluridine-tipiracil (4 vs. 2,5 months)
HERACLES-A	II	KRAS wild-type HER2-positive mCRC	35	Trastuzumab, lapatinib	PFS	Trastuzumab + Lapatinib (4.7 months)
MOUNTAINEER	II	KRAS wild-type HER2-positive mCRC	117	Trastuzumab, lapatinib	ORR	Tucatinib + trastuzumab (38.1%)
Fu et al	II	RAS/BRAF wild-type HER2-positive CRC	20	Pyrotinib, trastuzumab	ORR	Pyrotinib + trastuzumab (22.2%)
Yoshino et al. /DESTINY-CRC01	II	HER2-positive mCRC progressed after ≥2 prior regimens	86	Trastuzumab, deruxtecan	ORR, PFS, OS, DoR	ORR 45.3%, mPFS 6.9 moths, OS 15.5months, DoR 7.0 moths
MyPathway	IIa	HER2-positive mCRC	57	Trastuzumab, pertuzumab	ORR	ORR 32%
Hurwitz et al.	III	previously untreated mCRC	813	Bevacizumab	OS, PFS	IFL plus bevacizumab vs. IFL plus placebo (mOS 20.3 vs. 15.6 moths, mPFS 10.6 vs. 6.2 months)
Prager et al.	III	patients who had received two or fewer chemotherapy regimens in the advanced stage of CRC	246	Bevacizumab, tifluridine-tipiracil	OS, PFS	combination group vs. FTD-TPI group (mOS 10.8 vs. 7.5 months, mPFS 5.6 vs. 2.4 months))
RAISE	III	disease progression during or within 6 months of the last dose of first-line therapy of CRC	1,072	Ramucirumab	OS	ramucirumab vs. placebo (mOS 13.3 vs. 11.7 months)
AFFRIM	II	mCRC first line treatment	236	Alibercept	PFS	PFS at 12 months aflibercept/mFOLFOX6 vs. mFOLFOX6 (25.8% vs. 21.2%) mPFS aflibercept/mFOLFOX6 vs. mFOLFOX6 (8.48 vs. 8.77 months)
CORRECT	III	mCRC and progression during or within 3 months after the last standard therapy	760	Regorafenib	OS	Regorafenib vs. placebo (mOS 6.4 vs. 5.0 months)
IMblaze370	III	unresectable locally advanced CRC or mCRC	363	Regorafenib, atezolizumab, cobimetinib	OS	Atezolizumab plus cobimetinib vs. atezolizumab, regorafenib (mOS 8.87 vs. 7.10 vs. 8.51 months)
CONCUR	III	mCRC with at least two previous treatment lines or unable to tolerate standard treatments	243	Regorafenib	OS	Regorafenib vs. placebo (mOS 8.8 vs. 6.3 months)
FRESCO	III	mCRC that progressed after at least 2 lines of chemotherapy but had not received VEGFR inhibitor therapy	416	Fruquintinib	OS	Fruquintinib vs. placebo (mOS 9.3 vs. 6.6 months)
FRESCO-2	III	mCRC that received all current standard approved cytotoxic and targeted therapies and progressed on or intolerant to trifluridine–tipiracil or regorafenib	691	Fruquintinib	OS	Fruquintinib vs. placebo (mOS 7.4 vs. 4.8 months)

### 3.1 EGFR pathway

The erythroblastosis oncogene B (ERB) family consists of four transmembrane tyrosine kinase receptors: EGFR (ErbB1 or HER1), HER2 (ErbB2), HER3 (ErbB3), HER4 (ErbB4) (58). Overexpression of EGFR is one of the potential pathophysiological components of CRC as HER2 (1.3%–47.7%) and EGFR (25%–82%) overexpression have been widely reported among patients with CRC (57, 59, 60). Additionally, mutations within ERB may be accompanied by the increased tyrosine kinase activity existing separately or induced by the therapy (60). Clinically wise, overexpression of the receptors is considered to be correlated with poor prognosis and lower survival rate, metastases, drug resistance (61).

The chain of reaction of EGFR, HER3, and HER4 is induced by binding a ligand to their extracellular domains which allows the formation of active hetero-oligomers and phosphorylation of the tyrosine kinase domain (58). Activation of HER2 is still a matter of discussion as currently, the scientific community has not elucidated any ligand family that stimulates its extracellular domain. The more commonly accepted theory involves the formation of heterodimers with any of the other three receptors from the ERB family which creates a high-affinity complex for the ligands (62, 63). Particularly, HER3 receptor must undergo dimerization with the other ERB receptor due to lack of its intrinsic kinase activity. Regardless of ERB-receptor activation pathways, the initiated signalling pathway promotes proliferation, angiogenesis, continued existence, migration, and adhesion of the stimulated cells (56). Since cancer cells depend on those pathways, ERB family receptors play a crucial role in target treatment.

A list of typical adverse effects of anti-EGFR drugs includes: papulopustular rash, mucositis, xerosis, hypermagnesemia, diarrhoea, hypersensitivity/allergic reaction, skin fissuring, and paronychia infection (56, 64).

So far two antibodies have been registered for targeting EGFR: cetuximab and panitumumab (IgG2 antibody) as both of them are based on a similar mechanism—attachment to the extracellular domain of EGFR inhibiting binding of ligands which leads to tyrosine kinase internalization and degradation. Additionally, cetuximab binds to NK cells leading to antibody-dependent cellular and complement-mediated cytotoxicity (56, 60).

Panitumumab was registered based on a PRIME trial in which it was added to FOLFOX4 in first-line treatment markedly improving PFS (10 months vs. 8.6 months) in patients with wild-type KRAS CRC (65). Median OS was 23.9 months vs. 197 months. The acceptance of cetuximab was based on a CRYSTAL trial in combination with the FOLFIRI regimen (66). The previously untreated patients had 0.68 HR (95% CI, 0.50–0.94) compared to FOLFIRI alone (1.07). Median PFS was 9.9 months vs. 8.7 months in favour of the combination. It is essential to highlight that positive effects were observed only in patients with the KRAS wild-type variant.

In ASPECCT—a phase III RCT—panitumumab was found non-inferior to cetuximab as no differences in terms of OS, PFS, and ORR between these two drugs were found. As far as ADRs are concerned, the incidence of skin adverse effects was comparable. However, grade 3–4 hypomagnesemia was more frequent among patients treated with panitumumab, but grade 3–4 infusion reactions were more common in cetuximab. In other studies, patients with therapeutic plans based on panitumumab more often developed mutations in the EGFR extracellular domain than those treated with cetuximab but lower risk of developing hypersensitivity reactions (0.6%–3% vs. 3.5%–7.5%) (64, 67).

Left-sided CRC is characterized by higher expression of EGFR than their right-sided counterparts which has its clinical implications as shown during the Fire-3 trial which proved cetuximab is a superior treatment option for left-side CRC compared to bevacizumab when added to FOLFIRI (68). It depicted higher ORR (77% vs. 65%) and longer median OS (33 vs. 26 months) but PFS was comparable between the two groups. It is important to note that this advantage was observed only in left-side tumours.

EGFR inhibitors do not typically present a response in patients with BRAFV600E mutations when used as single agents or combined with cytotoxic chemotherapy. However, there is evidence that simultaneous inhibition of EGFR and BRAF can be beneficial in patients who underwent prior one or two regimens in refractory CRC. Kopetz et al. in the SWOG S1406 trial on 106 patients with BRAFV600E demonstrated the efficacy of a combination of irinotecan and cetuximab with the addition of Vemurafenib (69). The observed PFS of the group with all three medications vs. the group without vemurafenib was 17% vs. 4%. The DCR was 65% vs. 21% but OS was comparable between the two groups. Simultaneous blockade of EGFR and BRAF results in downregulation of mismatch-repair (70).

The combined treatment represented a more successful approach also in the study by (71) in which 44 patients received therapy with adagrasib and 32 adagrasib combined with cetuximab. The ORR was 19% vs. 46%, the median response duration was 4.3 months vs. 7.6 months and the PFS was 5.6 vs. 6.9 months—all in favour of combined therapy. Also, the grade 3 or 4 adverse effects rate was lower among the adagrasib-cetuximab group (34% vs. 16%).

Cetuximab monotherapy was also shown to be effective but, unsurprisingly, combined treatment has better clinical outcomes such as in the trial by (72). PFS of a group receiving cetuximab plus irinotecan was 4.1 vs. 1.5 months compared with a group receiving monotherapy, ORR 22.9% vs. 10.8% and median OS was 8.6 months vs. 6.9 months. A similar outcome was achieved during the ERMES phase 3 study (73) which resulted in reporting a lack of non-inferiority of cetuximab maintenance therapy compared to the FOLFIRI/Cet regimen. However, treatment with cetuximab alone presented a lower risk of grade 3 adverse effects rate.

Maintenance therapy is another field in which panitumumab has found its application (74). Currently, the main purpose in the development of maintenance therapy is the reduction or replacement of oxaliplatin-based chemotherapeutics due to high toxicity and tolerance issues. In the PANAMA trial, panitumumab in combination with folinic acid and fluorouracil was confronted with fluorouracil and folinic acid alone in patients with RAS wild-type CRC. There was noted a significant improvement in terms of PFS (8.8 months vs. 5.7 months) and also better outcomes in OS (28.7 months vs. 25.7 months), ORR (40.8% vs. 26%). These data suggest that adding panitumumab after induction to standard maintenance therapy is a viable clinical option. However, in the phase 2 VALENTINO trial panitumumab alone did not prove superior as a maintenance therapy over the panitumumab plus fluorouracil-leucovorin in terms of 10 months PFS (75).

Overall, cetuximab and panitumumab were shown as effective in first-line therapy of left-sided CRC and are recommended to be included in the treatment of RAS, BRAFV600E, and ERBB2 wild-type variants (57, 70). The major concern of anti-EGFR inhibitors is unsatisfying outcomes in the management of patients with mutated variants of CRC. However, some patients with KRAS-mutation G13-D mutation benefited from administering anti-EGFR therapy, suggesting that not all cases present resistance to this line of drugs (8, 76).

In second-line therapy and above, anti-EGFR agents are not typically among the top choices for the further management of mCRC as many studies failed to reach statistically significant results of their benefit (57). Nevertheless, some trials demonstrated their usefulness in particular clinical scenarios (66). In a phase III trial presented evidence for the efficacy of panitumumab in refractory CRC in a group of 463 patients with 1% or more EGFR tumour cell membrane staining. The outcomes of panitumumab with best supportive care vs. best supportive care alone were as follows: PFS (8 weeks vs. 7.3 weeks), ORR (10% vs. 0%) with equal OS.

Due to unsatisfactory available methods for third-line treatment of CRC, there are attempts to evaluate the effectiveness of panitumumab in rechallenge therapy of refractory RAS wild-type CRC. According to (77), mCRC cells develop resistance mechanisms, notably through RAS and EGFR ectodomain mutations, under sustained anti-EGFR treatment. However, upon cessation of this treatment, mutated malignant cells lose their competitive advantage over non-mutated malignant cells, leading to a decline in their numbers over time. Parseghian et al. elucidate that the relative mutant allele frequency of RAS and EGFR undergoes exponential decay (r2 = 0.93 for RAS; r2 = 0.94 for EGFR) with a cumulative half-life of 4.4 months which potentially rationalizes rechallenge and intermittent anti-EGFR therapy. In a trial by (78) 62 patients were divided into two groups and tested with tifluridine-tipiracil with or without panitumumab. Median PFS was noted as 4 months compared to 2.5 months. Furthermore, in patients with pretreatment plasma RAS/BRAF wild-type ctDNA, there was observed even better response to treatment. PFS of the group with this pattern of DNA and simultaneously receiving panitumumab was 4.5 months and PFS rates at 6 months were 38.5% vs. 13% (compared to a group that did not present RAS/BRAF wild-type ctDNA and received panitumumab).

As far as the intermittent therapy with an anti-EGFR agent is concerned, during the phase 2 trial IMPROVE (79) patients treated as a first-line treatment with FOLFIRI/PANI continuously not only had lower PFSot compared to the intermittent regimen (12.6 months (95% CI: 9.0–16.1) vs. 17.6 months (95% CI: 7.5–27.8) respectively) but also higher risk of grade 3 or 4 toxicities. Nevertheless, further evaluations of the results are required for a better understanding of intermittent therapy with anti-EGFR agents.

#### 3.1.1 HER2-inhibitors

Overexpression of HER2 represents a relatively small group of patients with CRC, being more commonly associated with breast or gastric cancers. Depending on the study it pertains from 2% to 11% of CRC cases, typically localized in the left side of a colon and the rectum. Its prognostic role is yet still unclear as some studies report a shorter time to recurrence and lower OS, the impact of HER2 in CRC seems to be not as meaningful as the other major gene alterations related to this tumour. The relatively small population of patients with this mutation is an evident obstacle in the evaluation of targeted treatment (80).

Currently, HER2 inhibitors are not included in routine use except for tucatinib and trastuzumab regimes which have been approved by the FDA in 2023 (81). However, clinical trials demonstrated a potential for upcoming targeted therapies focused on HER2-related pathways. In the HERACLES-A study combination of trastuzumab and lapatinib was administered to 35 patients with KRAS wild-type HER2-positive variant. In 6.7 years follow-up mPFS was 4.7 months, median OS 10 months and ORR was 28% which supports the use of mentioned drugs (82). In the MOUNTAINEER clinical trial, the same variant was treated with trastuzumab plus tucatinib which resulted in an ORR of 38.1%. Similar outcomes in ORR were achieved in comparison of patients who previously received anti-EGFR therapy vs. those who did not (36.4% vs. 40%) (81).

mCRC management with pyrotinib has been reported only for small cohorts of patients. In the phase II clinical trial by Fu et al. a group of 20 patients with RAS/BRAF wild-type HER2-positive CRC was evaluated in response to treatment with a combination of pyrotinib and trastuzumab (83). The mPFS was 4.3 months in the RAS-wild type group and 3.4 months in the overall population. Likewise, ORR was 33.3% and DCR 83.3% vs. ORR 22.2% and DCR of 61.1%. The same drug combination was used by Chang et al. with an ORR of 50% in the general population and 57.1% in RAS-wild-type patients. The general median PFS amounted to 7.53 months and the OS was 16.8 months (84). In a trial conducted by Zhou et al. who compared the results of the use of pyrotinib monotherapy vs. pyrotinib plus trastuzumab, PFS was at 5.5 months for a single drug and 8.6 months for a combination, ORR 25% and 50% respectively (85) OS was evaluable only in the monotherapy group and was 10.9 months. These results show potential for further studies due to observed antitumor activity.

In the phase 2 trial by Yoshino et al. combined therapy with the use of trastuzumab deruxtecan was applied to HER2-positive patients with prior at least 2 administered regiments (86) as 3 cohorts were assembled which only Cohort A noted an objective response rate (ORR) of 45.3%. Trastuzumab was also paired with pertuzumab in the MyPathway trial (87). The results of the examined group of 57 patients with refractory HER2-positive CRC were OR at 32% and grade 3 plus ADR at 37%.

In summary, a meta-analysis that compiled several key anti-HER2 targeted treatments conducted by Wang et al. confirmed the efficacy of this method resulting in a median PFS of 4.35 months, ORR of 27.5%, and DCR of 68.9%. Yet, it is worth noting that the incidence of all-grade adverse effects was estimated as 93.5% and grade 3 or higher at 16.8% (88).

### 3.2 Angiogenic pathway

The uncontrolled proliferation of cancer cells results in inadequate perfusion of tumour cells, giving rise to local acidosis and hypoxia which leads to the promotion of the transcription of hypoxia-associated factors, specifically HIF-1a and HIF-2a which subsequently initiates the transcription of several key ligands for angiogenesis e.g., VEGF (vascular endothelial growth factor), angiopoietin, Notch and integrins (89) hence angiogenesis is inherently linked with the process of carcinogenesis and creation of metastases.

The pathway of paramount clinical relevance involves the VEGF-A ligand and VEGFR-2 receptor, facilitating endothelial cell migration and proliferation, elevating vascular permeability, and inducing alterations in gene expression (90). Given its important contribution to the pathophysiology of carcinogenesis, numerous anti-VEGF agents have been developed, exhibiting notable success in the management of mCRC e.g., Bevacizumab, Ramucirumab, Aflibercept, and Regorafenib.

#### 3.2.1 Bevacizumab

Bevacizumab is a humanized monoclonal antibody that targets vascular endothelial growth factor A (VEGF-A) and subsequently interferes with its primary mechanism of action. This therapeutic agent exerts its effects through the direct neutralization of circulating VEGF-A. Notably, it also interferes with VEGF-A particles located in endothelial progenitor cells within the bone marrow, thereby inhibiting their proliferation and systemic migration. By binding to the extracellular domain of VEGF-A, bevacizumab effectively obstructs interactions between VEGF-A and KDR and Flt-1 receptors, which are present in both cancerous and unaltered endothelial cells (91). This dual receptor blockade is significant as it contributes to the inhibition of critical pathways involved in angiogenesis and tumour growth. In addition to its receptor-mediated effects, bevacizumab disrupts perfusion and permeability within cancerous tissues which occurs through a reduction in intestinal pressure, leading to altered vascular dynamics within the tumor microenvironment. Furthermore, bevacizumab exhibits pleiotropic effects on the immune system, influencing various facets of the body's defence mechanisms against cancer (92, 93).

Clinical studies, such as the trial conducted by Hurwitz et al., evaluated the efficacy of bevacizumab when added to standard therapy. In this trial, which included irinotecan, fluorouracil, and leucovorin (IFL), the addition of bevacizumab resulted in a significant improvement in PFS. The median PFS was extended to 10.6 months compared to 6.2 months in the group receiving IFL with a placebo. Additionally, the ORR favoured the bevacizumab group, with values of 44.8% compared to 34.8%. The duration of response (DRS) also demonstrated a substantial benefit for the bevacizumab-treated patients, with a DRS of 10.4 months as opposed to 7.1 months in the placebo group. The primary endpoint of the study was to establish the mOS, which further solidified the efficacy of bevacizumab. The bevacizumab group exhibited a prolonged mOS of 20.3 months compared to 15.6 months in the placebo group.

 (94) demonstrated the benefit of adding bevacizumab to the protocol involving trifluridine–tipiracil for patients who had received two or fewer chemotherapy regimens in the advanced stage of CRC, predominantly as third-line therapy for over 90% of patients. The combined treatment yielded an mOS of 10.8 months, surpassing the exclusive use of trifluridine–tipiracil, which resulted in an mOS of 7.5 months. Moreover, the mPFS for the combined treatment was 5.6 months, contrasting with the placebo group's mPFS of 2.4 months. The median time to worsening of the Eastern Cooperative Oncology Group (ECOG) performance status score was significantly extended in the combined treatment group, measuring 9.3 months, compared to 6.3 months in the exclusive trifluridine–tipiracil cohort. These findings highlight trifluridine–tipiracil plus bevacizumab as an effective therapeutic option for refractory CRC, irrespective of mutations, ADRs, or prior history of bevacizumab treatment. Similarly, bevacizumab demonstrated efficacy when combined with capecitabine in elderly patients, resulting in an extended OS of 20.7 months compared to 16.8 months. Additionally, in the first-line treatment of CRC, the addition of bevacizumab to FOLFOXIRI showed superior outcomes with an OS of 31 months, surpassing FOLFIRI alone, which achieved an OS of 25.8 months (95, 96).

Importantly, bevacizumab's effectiveness was consistent across patients with KRAS mutant and wild-type CRC, dispelling differences in treatment outcomes between RAS wild-type and RAS mutant subtypes (8).

#### 3.2.2 Ramucirumab

Ramucirumab, a human IgG1 anti-VEGF-A antibody with an additional inhibitory effect on vascular endothelial growth factor receptor-2 (VEGFR-2) (8) has demonstrated efficacy in second-line treatment when combined with FOLFIRI, as evidenced by the phase-3 RAISE trial (97) focused on a patient cohort that experienced progression during or after receiving treatment with oxaliplatin, fluoropyrimidine, or bevacizumab. In comparison to a placebo, the addition of ramucirumab resulted in notable improvements in OS (13.3 vs. 11.7 months) and PFS (5.7 vs. 4.5 months). Notably, adverse effects of grade 3 or worse were observed in more than 5% of patients (98, 99).

Exploring its potential in combination with triplet chemotherapy, ramucirumab exhibited promising outcomes as patients receiving second-line therapy with ramucirumab experienced better median to-treatment discontinuation and OS when compared to those receiving ramucirumab in the third-line or later stages. Specifically, the respective durations were 6.7 months vs. 3.6 months, and “not reached” vs. 7.6 months (100).

#### 3.2.3 Aflibercept

Aflibercept, also known as VEGF Trap, operates as a soluble agent by binding with multiple endogenous receptors and interrupting their function (101). Notably, aflibercept demonstrates a higher affinity and faster binding capability to multiple isoforms of VEGF-A compared to bevacizumab, and it uniquely targets VEGF-B, VEGFR1 ligands, and PIGF (placental growth factor). Its characteristics suggest potential efficacy in overcoming resistance developed after prior bevacizumab treatment Importantly, the therapeutic effectiveness of aflibercept does not appear to be influenced by the presence of KRAS or BRAF mutations or the patient's history of prior bevacizumab treatment (102).

In the context of CRC treatment, aflibercept is recommended for second-line therapy. When added to the FOLFIRI protocol in patients with a background of oxaliplatin treatment, aflibercept demonstrated significant improvements in OS (13.5 vs. 12.06 months), PFS (6.9 vs. 4.67 months), and RR (19.8% vs. 11.1%) (103). However, in the first-line setting, the AFFRIM study reported no difference in PFS between the combination of aflibercept with FOLFOX6 and FOLFOX6 plus placebo, although the combination with aflibercept was associated with a higher level of toxicity (104).

Real-world studies support aflibercept's safety profile, with hypertension, neutropenia, and gastrointestinal incidents being the most commonly observed adverse effects. Importantly, these effects are generally manageable, positioning aflibercept as a relatively safe treatment options in clinical practice (105).

#### 3.2.4 Regorafenib

Regorafenib, a kinase inhibitor, targets a spectrum of receptors involved in angiogenesis (TIE2, VEGFR 1–3), stromal signalling (FGFR, PDGFR-β), and oncogenic pathways (KIT, RAF, REF) (106). In the CORRECT trial, it was assessed as a last-line monotherapy for CRC against a placebo (107). The primary endpoint, OS, demonstrated superiority with regorafenib at 6.4 months compared to 5 months with placebo. PFS was also favourable at 1.9 months vs. 1.7 months, though adverse effects were more pronounced at 93% vs. 61%.

In the IMblaze370 trial, regorafenib was compared to the combination of atezolizumab plus cobimetinib in third-line therapy for microsatellite-stable CRC (108). While the combination exhibited a slightly longer median OS at 8.87 months vs. 8.51 months for regorafenib alone, the difference was modest. Grade 3 or 4 adverse effects were observed in 61% and 58% of patients, respectively. The CONCUR trial further supported regorafenib's efficacy, revealing an OS of 8.8 months vs. 6.3 months with a placebo and a PFS of 3.2 months vs. 1.7 months (109).

Despite its efficacy in refractory CRC, regorafenib's primary drawback lies in its adverse effects. In response (110), explored a dose escalation strategy vs. standard dosing to mitigate toxicity. The endpoint, the proportion of patients eligible and consenting for initiation of the third cycle, favoured the dose escalation group at 43%, compared to 26% in the standard dosing group. Adverse effects were reduced in the dose escalation strategy (13% vs. 18%) while maintaining comparable drug activity. Regorafenib's effectiveness in third-line treatment underscores its potential, but vigilant monitoring and management of adverse effects are imperative for optimal patient care.

#### 3.2.5 Fruquintinib

In 2023, the FDA approved fruquintinib, a highly selective oral inhibitor targeting VEGF receptors 1, 2, and 3, specifically for patients with refractory CRC. The FRESCO trial, comprising 416 patients, pitted fruquintinib against a placebo, revealing a significant increase in median PFS (3.7 months vs. 1.8 months) and mOS (9.3 months vs. 6.6 months) (111). This promising outcome was corroborated in the FRESCO-2 trial, which enrolled 691 patients previously subjected to all standard targeted and cytotoxic therapies, demonstrating progression or intolerance to regorafenib or trifluridine-tipiracil. Notably, the median number of prior lines of treatment was 4. In this trial, fruquintinib notably improved median OS at 7.4 months compared to 4.8 months in the placebo group. However, the rate of grade 3 or worse adverse effects was higher in the fruquintinib group (63%) compared to the placebo group (50%) (112).

## 4 Other potential treatment targets for personalized therapy

Each CRC case exhibits a variable mutation count ranging from 60 to 1,500 mutations, with only a subset holding clinical significance (113, 114). The recurrent mutations in mCRC involve APC, TP53, KRAS, and PIK3CA, impacting critical signalling pathways like MAPK, WNT, PI3 K, TGF-β, and p53, crucial in mCRC tumorigenesis. The WNT pathway, especially, plays a pivotal role, in influencing β-catenin regulation through ubiquitin-mediated degradation and phosphorylation, contributing to cancer stem cell renewal, proliferation, and differentiation. Several molecular drug targets have significantly advanced clinical progress in targeted and immunotherapies (115, 116).

### 4.1 NTRK fusions

The NTRK genes (NTRK1/2/3) encode tropomyosin receptor kinase proteins (TRK A/B/C) primarily involved in neural development and cellular homeostasis (117). NTRK fusions are a rare occurrence in CRC patients, accounting for around 1% of cases (118–120). Previous reports have indicated that CRC characterized by NTRK positivity may constitute a distinctive subset exhibiting specific features e.g., high TMB and a higher likelihood of MSI (121).

Recent trials—ALKA-372-001 and STARTRK, evaluating TRK inhibitors, such as larotrectinib and entrectinib, are prompting clinicians to consider NTRK testing in mCRC patients, particularly in later lines of therapy (122, 123). Larotrectinib is a first-in-class, highly selective TRK inhibitor, which produces durable responses in patients with NTRK fusion–positive, according to long-term data from the phase II basket of NAVIGATE trial (NCT02576431). Out of 34 patients treated with larotrectinib, 33% showed an OR (3% complete response, 30% partial response) (124). Additionally, 45% had stable disease, 12% experienced disease progression, and 9% had an undetermined best overall response. Notably, 9 out of 29 patients with measurable disease experienced tumour shrinkage.

To overcome secondary resistance mechanisms, ongoing clinical evaluations are assessing the efficacy of additional second-generation TRK inhibitors, including repotrectinib and taletrectinib (125).

### 4.2 FGFR

The FGFR1-4 (fibroblast growth factor receptor) genes encode four closely related receptor tyrosine kinases, which compose the FGFR family. After binding with FGF (fibroblast growth factor) ligands the RAS/MAPK, PI3 K/AKT, and JAK/STAT pathways are activated (126). The FGFR signalling pathway is involved in a range of biological functions spanning from angiogenesis, wound repair, and tissue regeneration to cell proliferation, migration, and anti-apoptotic processes. Among individuals diagnosed with CRC, around a third of them present modifications in FGFR genes, involving specific mutations, amplifications in gene copy numbers, and heightened mRNA expression levels (127). Prior preclinical studies indicated that these genetic modifications were linked to sensitivity to FGFR inhibitors, but predicted a poor prognosis, invasiveness, and the potential for metastases (128).

Over recent years, various targeted FGFR inhibitors have undergone clinical trials to assess their therapeutic impact on patients. These compounds competitively attach to the ATP-binding sites of FGFR interrupting further signal propagation. However, due to their high specificity, broad applicability in treating CRC patients is impossible. Several TKIs have been used to treat tumours with FGFR irregularities. Ponatinib, dovitinib, and lucitanib have undergone assessment for their ability to inhibit FGFR, yet their high toxicity has limited their ability for future development (127). As of late, F1-7, a new FGFR inhibitor has been outlined, which can induce DNA damage. This results in impeding cell growth and metastasis, ultimately causing cellular apoptosis. Experiments conducted on mouse models further validated the effective suppression of tumour growth by inhibiting the FGFR pathway (129).

Pemigatinib, another inhibitor of FGFR1-3, during a phase II single-arm study, showcased improved response rates among patients dealing with resistant metastatic CRC linked to FGF/FGFR alterations. All participants enrolled in the trial and treated with pemigatinib exhibited confirmed mutations in FGFR1-4 and/or amplifications in FGF/FGFR, with no FGFR translocations. The mPFS stood at 9.1 weeks (95% CI, 7.9 to not evaluable [NE), while the mOS reached 7.9 months (95% CI, 3.4 to NE). Severe grade 3 or higher adverse events (AE) were observed in 42.9% of treated patients, including one instance of a grade 5 AE. Among the most frequently occurring AE of any grade were anaemia, hyperphosphatemia, elevated alkaline phosphatase, increased AST levels, and fatigue. Despite a favourable safety profile, pemigatinib demonstrated suboptimal effectiveness in this specific population. Ongoing translational studies aim to unravel the mechanisms contributing to resistance against pemigatinib (130).

### 4.3 DNA damage repair genes

The DNA damage response (DDR) pathway plays a vital role in identifying and accurately repairing damaged DNA, crucial for preserving the cell's genetic integrity and preventing issues like cellular ageing, apoptosis, and the onset of cancer. This pathway involves eight distinct processes that cater to different types of DNA damage, including mismatch repair (MMR), base excision repair (BER), nucleotide excision repair (NER), homologous recombination repair (HRR), nonhomologous end-joining (NHEJ), checkpoint factors (CPF) and more (131). DDR alterations can prompt a hyper-mutated state or microsatellite instability-high (MSI-H), leading to increased TMB, which acts as a biomarker predicting better responses to immune checkpoint inhibitor (ICI) therapy (132).

Recent studies have unveiled germline and/or DDR defects in a subset of CRC cases, ranging from 13.8% to 36% prevalence (133). These alterations, irrespective of microsatellite instability status, were linked to a higher median tumour mutation burden in CRC, along with increased positivity for PD-L1. Moreover, DDR mutations have shown an association with enhanced overall survival (OS) in CRC patients undergoing treatment with immune checkpoint inhibitors (ICIs). Notably, investigations suggest that DDR-related ATM or BRCA2 somatic mutations hold promise as biomarkers for evaluating the response of stage III CRC patients to oxaliplatin-based chemotherapy (134). In a study performed by (135), DNA DDR mutations were identified in all MSI-H CRCs and 83.77% of MSS CRC cases. The most frequently mutated DDR genes included ARID1A (7.5%), ATM (5.7%), and BRCA2 (2.6%).

The predominant mutation type seen in ARID1A, characterized as a truncating mutation, induces deficiencies in DNA damage repair mechanisms within tumour cells (136). Preclinical research has demonstrated that this deficiency in ARID1A makes CRC cells more responsive to PARP inhibitors (like olaparib, rucaparib or veliparib) both in laboratory experiments and in animal models (137).

Comparison between right- and left-sided CRCs revealed no notable variance in DDR genes and associated pathways. Survival analysis indicated that DDR mutations did not correlate with OS in MSS CRCs. However, left-sided CRC patients with mutations in the homologous recombination repair (HRR) pathway displayed notably prolonged OS compared to right-sided CRCs. Additionally, mutations in the DDR pathway, including those within the homologous recombination repair (HRR) pathway, didn't significantly correlate with improved OS in MSS CRC patients (135). Conversely (133), reported a strong association between DDR mutations and MSI, noting a favourable mOS in CRC patients treated with immune checkpoint inhibitors (ICI). However, in their study, conventional treatments did not display a significant difference in prognosis for patients with DDR mutations, suggesting that DDR mutations might specifically serve as a predictive biomarker for the effectiveness of ICI immunotherapy in CRCs. Consequently, for MSS CRC, it appears that DDR mutations are not notably linked to a better prognosis.

Marks et al. (138) study aimed to explore the potential impact of DDR mutations on the response to first-line treatments containing oxaliplatin (FOLFOX/XELOX) or irinotecan (FOLFIRI) in mCRC. DDR mutations were identified in 11 out of 49 patients (22%). Among these cases, patients treated with a first-line oxaliplatin-based regimen exhibited a statistically significant improvement in mOS (3.4 vs. 1.8 years; *P* = 0.042) compared to those receiving irinotecan. Additionally, this group showed a numerically higher response rate (50% vs. 33%; *P* = 0.58). However, in patients without DDR mutations, no significant differences in OS (2.4 vs. 2.5 years; *P* = 0.42), response rate, or disease control rate were observed between the two regimens.

### 4.4 POLE

The POLE gene encodes an enzyme called DNA polymerase epsilon, which holds an important role in DNA replication fidelity. Responsible for synthesizing the leading DNA strand at the replication fork, POLE encompasses a 3′-5′ exonuclease domain crucial for enhancing the precision of replication. This domain functions by identifying and excising mismatched base pairs, thereby promoting accurate DNA replication. Somatic and heritable deficiencies in POLE's proofreading mechanism, notably mutations occurring within its exonuclease domain, are more prevalent in tumours proficient in mismatch repair and cause a rise in the rate of mutations, which ultimately results in the development of tumours (139).

The occurrence rate of acquired mutations within the exonuclease domain of POLE in CRC stands at around 3%, surpassing the frequency observed in inherited mutations (“Comprehensive Molecular Characterization of Human Colon and Rectal Cancer,” 2012). While the prevalence of inherited mutations within the exonuclease domain of POLE in familial colorectal adenoma or CRC is merely 0.1%–0.25%, the presence of these germline mutations substantially amplifies the likelihood of developing CRC (140).

POLE somatic mutations are linked to a favourable prognosis and are more prevalent in men, the right colon, and individuals in the early stages of the disease (141). CRC patients carrying POLE mutations often showcase elevated TMB and infiltration of immune cells within their tumours, thereby amplifying their responsiveness to immune checkpoint inhibitors (ICI) (142).

In a study performed by (139) among 14,229 next-generation sequencing reports, 458 patient tumours exhibited POLE mutations. Of these mutations, 15.0% were categorized as pathogenic, 15.9% as benign, and 69.1% as variants of unknown significance. Among 82 patients who received programmed death 1 or programmed death ligand-1 inhibitors either individually or alongside cytotoxic T-cell lymphocyte-4 inhibitors, those with pathogenic POLE mutations demonstrated substantial improvements. They exhibited enhanced clinical benefit rates (82.4% vs. 30.0%; *P* = .013), a longer median progression-free survival (15.1 vs. 2.2 months; *P* < .001), increased overall survival (29.5 vs. 6.8 months; *P* < .001), and a prolonged treatment duration (median 15.5 vs. 2.5 months; *P* < .001) in comparison to individuals harbouring benign variants.

The role of immunotherapy among patients with POLE mutation remains unclear, however, in 2020, a prospective, open-label, multicenter phase 2 experiment was carried out by (143) to evaluate the safety and efficacy of avelumab in 30 patients with dMMR/MSI-H and 3 patients with POLE mutations. Even though no patients with POLE mutations responded to avelumab, it is important to consider the constraints of the small sample size and the variance in mutation locations.

### 4.5 RET

RET is a proto-oncogene that produces the receptor for growth factors from the glial-derived neurotrophic factor family (144). Abnormal chromosomal rearrangements can result in RET fusion, which is most often observed in thyroid and lung cancers, however, in rare instances, RET gene fusion can also occur in mCRC cancer, with a frequency of less than 1% (145). mCRC tumours with in-frame RET display unique characteristics, such as a preference for the right colon, diagnosis at an older age, and predominantly MSI-h phenotype (146).

One oral RET inhibitor, selpercatinib, was approved by the FDA in 2020 to treat RET fusion-positive non-small cell lung cancer and thyroid cancer (147). In the phase I/II LIBRETTO-001 study, which included 41 patients with various solid tumours (excluding lung and thyroid cancer) (148), 10 patients (24%) had CRC. The ORR for these malignancies was 20% (95% CI, 2.5–56), while the OR in the entire group was 44% (95% CI, 28–60). People with solid tumours studied had a 5-year survival rate ranging from 3% to 40%. Another RET inhibitor, pralsetinib, was examined in the phase I/II ARROW trial. In this cohort of 23 participants, two had CRC, but no response was observed in these patients (149).

### 4.6 RSPO fusions and RNF43 mutations

The RSPO family consists of four genes RSPO1-4, which encode signalling proteins, known to play a significant role in activating the Wnt/β-catenin signalling pathway (150). This pathway is crucial for controlling important biological functions like cell growth, stem cell management, and maintaining tissue balance and regeneration. RNF43 is recognized as a negative modulator of WNT signalling and acts as a tumour suppressor (151). When RNF43 is lost, there is a reduction or absence of frizzled receptor degradation, leading to heightened WNT signalling. In cancer cells, the inactivation of RNF43 due to mutations is among the factors contributing to the sustained activation of the WNT signalling pathway.

The RSPO family is observed in up to 8% of CRC, and studies indicate that similar to RNF43 mutations, RSPO translocations, even in isolation, are sufficient to initiate carcinogenesis. RNF43 mutations, occurring in various malignancies including colorectal and gastric cancers, can be observed with a frequency of up to 20% (152). Notably, the frequency of RNF43 mutations is higher in MSI cancers. Neoplastic cells with loss-of-function mutations in RNF43 result in a higher quantity of the Wnt receptor Frizzled which, consequently, exhibits heightened sensitivity to the inhibition of the porcupine homolog (PORCN) protein, known for its role in posttranslational modification of the Wnt protein (153).

In a study performed by (154) the initial group of 7,245 CRC samples was examined. Among these, RSPO gene fusions (RSPOfp) were found in 1.3% of cases, while RNF43 mutations were present in 6.1%. Tumours with RNF43 mutations were notably linked to tumours located on the right side. Interestingly, none of the RSPOfp tumours displayed RNF43 mutations. RNF43-mutated tumours tended to have a higher occurrence of MSI-H/dMMR in 64.3% of cases and a tumour mutation burden of ≥10 mutations per megabase (mt/Mb) in 65.8%. In contrast, RSPOfp did not exhibit any correlation with MSI-H/dMMR.

The outcomes in the (155) study imply that RNF43 mutations are associated with better OS in CRC patients treated with ICBs, likely due to increased TMB and the presence of gene signatures related to the immune system.

The changes observed in RSPO2 expression and rearrangements offer a potential avenue for creating targeted treatments in tumours reliant on the Wnt pathway. Despite significant efforts, the availability of FDA-approved drugs and inhibitors for routine inclusion in clinical trials aiming at the Wnt pathway in CRC remains limited. Developing precise and effective inhibitors to target Wnt signalling in CRC continues to present significant obstacles (156).

Multiple ongoing clinical trials aim to validate the effectiveness of diverse inhibitors targeting the Wnt/β-catenin signalling pathway in RNF43/RSPO-positive tumours. Additionally, the identification of MSI-h/dMMR traits in a subset of RNF43-mutated tumours suggests that combining immune checkpoint inhibition with and without Wnt/β-catenin signalling inhibitors might represent a viable therapeutic strategy that should be assessed in future prospective trials (154).

## 5 Adoptive cell therapy

Adoptive cell therapy is a type of immunotherapy which involves allo- or autogenic genetically altered lymphocytes (157, 158). The immunologically active effector cells are being developed and modified *in vitro* yielding anti-tumor activity against particular host tumour cells (159). Currently, three types of ACT are used or undergo research in the field of oncology: T-cells with a chimeric antigen receptor (CAR-T), CD8 + tumour-infiltrating lymphocytes (TILs) and T-cell receptor-engineered T-cells (TCR-T) (158). The mechanism of TCR varies depending on the used type of cells. TCR based on cytotoxic T-cells enhances their ability to kill abnormal cells of a host and TCR related to regulatory T-cells inhibits the specificity and responsiveness of the immunologic system against a particular antigen (157, 160). CAR-T has been so far successfully used in hematologic malignancies such as leukaemia, multiple myeloma and lymphomas however, their utility in solid tumours is still debatable (159, 161). Nevertheless, several preclinical studies were conducted aimed at evaluating neoantigens or tumour-associated proteins which are common in mCRC such as carcinoembryonic antigen (CEA), guanylate cyclase 2C (GUCY2C), mesothelin (MSLN), human epidermal growth factor receptor-2 (HER2), epithelial cell adhesion molecule (EpCAM), doublecortin-like kinase 1 (DCLK1), natural killer group 2 member D ligand (NKG2DL), CD133, mucin 1 (MUC1) (162). The extent of ADRs and the effectiveness of CAR-T treatment predominantly depend on the target antigen specificity through which it is designed (163). The mild adverse effects which were present in patients with CRC include fever, anorexia, and fatigue. These were directly linked with an excessive cytokine release (163). TCR therapy against CEA was reported as inducing severe transient colitis. Presumably as a result of the expression of this antigen in normal colonic mucosa (165).

### 5.1 NKG2D

NKG2D is a type of immunoreceptor present in several subtypes of lymphocytes. It was proposed that it could serve as a target in the CAR-T type of therapy called CYAD-01. Physiologically the protein is either absent or its expression is relatively low but during infections or hyper-proliferation, there can be a significant elevation of its expression which allows the immune system to eliminate the affected cells. Its elevation was linked with poor survival in patients with most variants of CRC in contrast to other cancer types where its protective role was observed. This mechanism was used in adoptive cell therapy (166, 167). So far in-vitro and in-vivo experiments demonstrated cytotoxicity against CRC cells and significantly reduced the extent of the tumour and suppression of its growth in the xenograft model (168). The same outcome was observed in human subjects. In a study by Xiao et al., the modified NK cells were used in the therapy of 3 patients with CRC (163). In two patients, the infusion into the peritoneal cavity resulted in a reduction of tumour cells in intraperitoneal fluid and a reduction of ascites generation. In one patient USG-guided intrahepatic infusion allowed rapid regression of metastasis in the liver confirmed in a PET-CT scan.

### 5.2 CEA

CEA is an immunoglobulin-glycoprotein involved in cell adhesion (169), frequently found in the epithelium of the embryo which is also associated with overexpression in 98.8% of CRC tissue samples, making CEA the predominant prognostic marker for CRC (170). A pre-clinical study conducted by (171) concerning a novel, chimeric SIRPg-CD28 co-receptor for CRC exhibited anti-tumour *in vivo* properties. The cytotoxicity effect was specific only to CEA and CRC cells. In mice CRC xenografts models, the tumours were eradicated within 21 days and persisted until the end of the experiment. CAR-T with an “armed” co-receptor demonstrated remarkable anti-tumour efficiency in CRC.

In a phase-I study by (172), 10 patients received CEA-targeted CAR-T with progressing doses (1 × 105–1 × 108 /CAR + /kg cells). 7 of them noted transformation from progressive to stable disease, 2 of them remained stable after 30 weeks and 2 presented tumor shrinkage in imaging studies. No severe adverse effects were observed.

### 5.3 GUCY2C

GUCY2C is a cancer mucosa antigen expressed in both human and mouse intestinal mucosa. Similarly, to CEA, its overexpression in CRC implies its distinctive suitability as a potential target for CAR-T cell therapies. An open-label, single-arm, investigator-initiated exploratory trial conducted by (173) examined 13 individuals with GUCY2C-positive mCRC. In the analysis of 10 evaluable patients, there were 6 partial responses, 2 stable diseases, and 2 progressive diseases. Across the dosage levels, ORR = 60%, DCR = 80%. The primary toxicity was decreased lymphocytes in 12/13 pts (92%), leukopenia in 1/13pts and thrombocytopenia in 1/13pts. Overall, the anti-GUCY2C CAR-T cell therapy was tolerated well with mild AE.

### 5.4 HER2

Another potential target for CAR-T is HER2 transmembrane glycoprotein which as mentioned above exhibits tyrosine kinase activity playing a crucial role in regulating growth and differentiation of epithelial cells. The heightened expression of HER2 is observed in various types of solid tumours such as breast cancer, ovarian cancer, lung cancer, gastric cancer, and CRC (174), however, a mutation within the gene encoding the HER2 receptor is present only in 3–5% of mCRC (175) thus narrowing usefulness of target and CAR therapies aimed at HER2. The meta-analysis by (176) findings indicates that, on average, participants in the six trials, totalling 238 patients with HER2 + mCRC, had undergone a median of three prior lines of therapy before enrollment with ORR = 31.33% and DCR = 74.37%, PFS = 6.2 months. The outcomes suggest a significant response to the therapy, leading to meaningful enhancement in survival rates. Regarding CAR-T therapy for HER2 + mCRC, additional studies are required to elucidate its potential role in the future (177) observed that HER2 CAR-T cells demonstrate anti-tumour activity in CRC xenograft models. However, clinical trials are fraught with uncertainties. Phase I/II NCT00924287 clinical trial was terminated due to the first patient death because of treatment. However, TAC T-Cell therapy for HER-2 + solid tumours (NCT04727151) has been reported to be well tolerated without reported neurotoxicity in phase I, the only AE were haematological (178).

### 5.5 EpCAM

EpCAM, a prominent tumor-associated antigen found on the surface of CRC, has also been considered a potential target in CAR-T cell therapy which led to the development of clinical trials (NCT02915445, NCT03013712, NCT05028933, NCT03563326). First-in-human trial by (179) IMC001 designed to target EpCAM-positive gastrointestinal tumours with CAR-T has already exhibited promising anti-tumour capabilities and a favourable safety profile. Moreover, downsizing the tumour creates additional opportunities for surgical resection.

## 6 Management of mCRC among elderly patients with immunotherapy and targeted therapy

mCRC is predominantly observed in older adults, with 56% of incidence and 68% of mortality occurring in patients over 65 years old (180).

The treatment of elderly patients with mCRC includes systemic chemotherapy, targeted therapy, and in certain cases surgery or radiotherapy. Therapy should be individualized to be the most suitable for patients while aligning with the patient's general condition and preferences (8).

The majority of adults with mCRC are not candidates for surgical resection and instead require systemic therapy which is a reflection of an increased rate of comorbidities, frailty, and higher risk of cardiovascular or respiratory disorders among said population. The choice of systemic therapy should be guided by molecular profiling and mutational status, including RAS and BRAF mutations, microsatellite instability status, and primary tumour localization (181).

The first line of treatment is chemotherapy, which demonstrated the ability to significantly prolong a patient's lifespan by a factor of four (182). The foundation of most systemic treatments are fluorouracil (5-FU)-based regimes such as (183) FOLFOX or FOLFIRI. If the patient's deteriorated condition requires it, a reduced dose of 20% of FOLFOX or FOLFIRI can be administered (184, 185). This treatment can still be supplemented with oxaliplatin or irinotecan, but their effectiveness is controversial (184, 186).

As far as the targeted therapy is concerned among the elderly population, bevacizumab is the only VEGF inhibitor studied among elderly patients with mCRC in the AGITG MAX trial (187) as researchers demonstrated significantly improved PFS in geriatric patients after combining standard chemotherapy regime with bevacizumab. Also, the values of OS, RR, and PFS revealed no association with age.

Immunotherapy has also been introduced for elderly patients e.g., pembrolizumab, nivolumab, and ipilimumab (188). Although currently immunotherapy is only dedicated to patients with MSI-H tumours, the study of Aparicio et al. concluded that the older population has higher rates of mCRC associated with MSI-H, which can be seen in over 20% of cases (189). Studies on the safety of PD-1 inhibitors showed that these drugs are well tolerated in elderly patients with malignancies and their side effects are less severe than chemotherapy (190).

Similar effects were obtained with anti-EGFR drugs, which showed the outcomes of cetuximab did not differ from the younger patients with mCRC exhibiting low toxicity (191). Moreover, anti-EGFR treatment did not show any serious side effects, and combined with chemotherapy can be the option for initial treatment for elderly patients with mCRC (192). However, there are no randomized clinical trials among the elderly population, which could evaluate the efficiency of cetuximab and panitumumab compared to chemotherapy. Other therapeutic options such as pegorafenib, an oral multikinase inhibitor, are associated with poor therapeutic effects or serious side effects that can even result in death (193, 194).

## 7 Discussion

There is no doubt that novel therapies involving enhancing the immune system or based on inhibition of certain cellular pathways involved in the pathophysiology of malignancy are providing palpable results among mCRC patients as currently, the landscape of mCRC treatment has evolved with the integration of targeted therapies which is reflected by the wide usage of first-line treatments commonly involving standard chemotherapy regimens (FOLFOX, FOLFIRI, FOLFOXIRI) combined with anti-EGFR or anti-VEGF agents (8, 70, 158). Notably, the combination of bevacizumab with FOLFOXIRI stands out as the most effective regimen, achieving an outstanding OS of 31 months (96) compared to 19.5 months of OS among patients treated with FOLFOX (5-fluorouracil, leucovorin, and oxaliplatin) regime.

Although enormous efforts have been made in the last 20 years in the fields of targeted and immunotherapy, we still lack reliable methods for overcoming resistance and predicting the efficacy of the abovementioned therapies (8). Furthermore, the rationale of targeted therapy might be under critique since cost-to-benefit is in favour of standard chemotherapy as targeted therapy and costly genetic diagnostics offer only longer overall survival counted in months. Moreover, as mentioned by (57) targeted therapy also tends to induce grade 3 or 4 adverse effects which can be exacerbated when multiple agents are added to the treatment regimen.

With an ever-increasing understanding of genomic alterations among mCRC patients, there is a promising perspective in exploring novel approaches from recent advancements in genetics and immunology such as the development of therapies focused on changing the immunological phenotype of mCRC e.g., radioimmunotherapy (195) or photoimmunotherapy (196) holds promise for a broader application of immunotherapy in treating mCRC. Additionally, the potential benefits extend to adoptive cell therapy, small-molecule drug treatments (197), and leveraging recent advancements in nanotechnology (198). Progress in comprehending the role of microbiota in mCRC (199, 200) may contribute to further improvements in overall survival rates. Despite these advancements, it remains our opinion that achieving full recovery from mCRC still requires significant breakthroughs in our treatment approaches.
